# Novel Biomarkers for Contrast-Induced Acute Kidney Injury

**DOI:** 10.1155/2014/568738

**Published:** 2014-05-29

**Authors:** Carlo Briguori, Cristina Quintavalle, Elvira Donnarumma, Gerolama Condorelli

**Affiliations:** ^1^Laboratory of Interventional Cardiology and Department of Cardiology, Clinica Mediterranea, Orazio 2, 80121 Naples, Italy; ^2^Department of Molecular Medicine and Medical Biotechnology, Federico II University of Naples and IEOS CNR, 80131 Naples, Italy; ^3^IRCCS SDN Foundation, 80143 Naples, Italy

## Abstract

Biomarkers of acute kidney injury (AKI) may be classified in 2 groups: (1) those representing changes in renal function (e.g., serum creatinine or cystatin C and urine flow rate) and (2) those reflecting kidney damage (e.g., kidney injury molecule-1 (KIM-1), neutrophil gelatinase-associated lipocalin (NGAL), interleukin-18, etc.). According to these 2 fundamental criteria, 4 subgroups have been proposed: (1) no marker change; (2) damage alone; (3) functional change alone; and (4) combined damage and functional change. Therefore, a new category of patients with *“subclinical AKI”* (that is, an increase in damage markers alone without simultaneous loss of kidney function) has been identified. This condition has been associated with higher risk of adverse outcomes (including renal replacement therapy and mortality) at followup. The ability to measure these physiological variables may lead to identification of patients at risk for AKI and early diagnosis of AKI and may lead to variables, which may inform therapeutic decisions.

Contrast-induced acute kidney injury (CI-AKI) is associated with a prolonged in-hospital stay and represents an independent predictor of unfavorable outcome [1]. Therefore, it has been recommended to monitor renal function in all patients at risk with serial measurements of serum creatinine (sCr) following contrast media (CM) exposure [1, 2]. A rise in sCr or a reduction in urine output is the current golden standard for recognizing AKI [3]. However, the delayed increase in sCr is a potential reason for overlooking CI-AKI [4, 5] and, on the contrary, for prolonging hospital stay in the vast majority of patients who will not develop CI-AKI. In the last years, several studies investigated the significance and clinical utility of new biomarkers of kidney damage (Table 1). It has been proposed to classify biomarkers in 2 groups, namely, (a) those representing changes in renal function (e.g., serum creatinine or cystatin C and urine flow rate) and (b) those reflecting kidney damage, (e.g., kidney injury molecule-1 (KIM-1), neutrophil gelatinase-associated lipocalin (NGAL), interleukin-18, etc.). The conceptual framework of physiological biomarkers is superimposed upon the conventional clinical phases of acute kidney injury. A combination of kidney functional and damage markers simultaneously provides an easy method to stratify patients with AKI. According to these 2 fundamental criteria, 4 subgroups have been proposed: (1) no marker change; (2) damage alone; (3) functional change alone; and (4) combined damage and functional change [6] (Figure 1). Therefore, a new category of patients with “*subclinical AKI*” (i.e., an increase in damage markers alone without simultaneous loss of kidney function) has been identified. This condition has been associated with higher risk of adverse outcomes (including renal replacement therapy and mortality) at followup [7–9]. Thus, physiological biomarkers are not only needed in the early phase of AKI but also needed throughout the continuum of AKI. The ability to measure these physiological variables may lead to identification of patients at risk for AKI and early diagnosis of AKI and may guide therapeutic decisions. These physiological processes represent an integrative environment for the interaction of inflammatory mediators, imbalance in the homeostasis of oxygen, nitric oxide and oxygen radicals causing microcirculatory dysfunction, and impaired tissue oxygenation leading to AKI.

*Serum Creatinine as a Marker of CI-AKI.* Although in 80% of CI-AKI cases sCr starts rising within the first 24 h following CM exposure [10], the sCr typically peaks 2–5 days after CM and returns to baseline or near baseline within 1–3 weeks [1]. Therefore, in all patients at risk, a follow-up sCr should be obtained at 48–72 h following CM exposure [1, 2, 4, 11]. This implies an intrinsic delay of treatment of patients who will develop CI-AKI and, on the contrary, a prolonged hospital stay of patients who will not develop CI-AKI. sCr increase indicates a functional change (deterioration) not a damage (injury) of the kidney. Therefore, sCr will increase only in case of loss of function. Also creatinine suffers from two significant limitations [4]. First, creatinine excreted in the urine is not solely a result of glomerular filtration but also a result of renal tubular secretion [12]. This means that changes in sCr will underestimate the true fall in glomerular filtration rate (GFR). Second, following an acute fall in GFR, less creatinine is excreted. The retained creatinine is distributed in total body water. Thus, the serum level can be expected to rise slowly and will continue to rise until a new steady state has occurred. Therefore, although the injury induced by CM impairs GFR almost immediately, it requires 24–48 h for the fall in GFR to be reflected in an elevated level of sCr.

*Serum Cystatin C as a Marker of CI-AKI.* Cystatin C (CyC) is a 120-amino-acid, nonglycosylated protein that is a member of the family of cysteine proteinase inhibitors [13]. It is produced at a constant rate by all nucleated cells representing in the true sense of the word a “housekeeping gene product” [14]. CyC concentration is independent of age, sex, changes of muscle mass, and nutrition. CyC levels are lower in the hypothyroid and higher in hyperthyroid state as compared with the euthyroid state [14]. It is found in relatively high concentrations in many body fluids, and its low molecular weight (13.3 kDa) and positive charge at physiologic pH levels facilitate its glomerular filtration. It is later reabsorbed and almost completely catabolized in the proximal renal tubule [13]. Because of its constant rate of production, its serum concentration is therefore determined by glomerular filtration. Indeed, CyC does not undergo tubular secretion and appears in the urine solely through filtration [15, 16]. For these reasons, CyC has the potential to be a useful marker in detecting both chronic and acute changes in GFR [17–19]. The shorter (1.5 hours) half-life of CyC compared to creatinine accounts for the more rapid rise and the earlier attainment of a new steady state [20]. CyC is distributed in the extracellular volume [21], whereas sCr is distributed in the total body water [22], a volume which is 3 times larger. Therefore, the half-life of creatinine compared to CyC will be 3 times longer and the time to achieve a new steady state will increase proportionally implying that sCr will rise more slowly. It has been reported that (1) a CyC increase <10% at 24 hours is a reliable marker for ruling out CI-AKI and (2) a CyC increase ≥10% at 24 hours is an independent predictor of 1-year major adverse events (MAE), including death and dialysis [23]. The first observation may allow physicians an earlier discharge of the majority (>80%) of patients, thus avoiding an unnecessary prolonged hospitalization with associated practical and economic advantages [24]. The second observation identifies a subgroup of patients at higher risk for future MAE [25–27]. This observation may be explained by two reasons. First, CyC seems to be a better measure of kidney function than sCr and GFR [16, 24, 28]. Second, CyC may provide prognostic information beyond its role as an index of kidney function and, also, may be a better overall measure of the spectrum of pathophysiologic abnormalities that accompany kidney disease [29–31].

*Biomarkers of Kidney Damage.* Several studies have reported the ability of new biomarkers in both the earlier diagnosis of AKI and the robust prognostic significance [6, 7].

At present, utilization of the new biomarkers of kidney damage has been limited by several reasons: (a) the identification of the best biomarkers for each purpose (risk assessment, diagnosis, differential diagnosis, and prognosis), (b) uncertainty on the threshold (that may be different in each setting), (c) limited clinical evidence, and (d) costs [6, 32]. The characteristics of the ideal biomarker of CI-AKI are summarized in Table 2.

Limited evidence exists on* KIM-1*. This is a transmembrane protein not expressed in normal kidney but upregulated in dedifferentiated proximal tubule cells after ischemic or nephrotoxic AKI [33, 34]. KIM-1 was elevated to a much higher degree in patients with ischemic acute tubular necrosis than in patients with CI-AKI. Increased urinary levels have been reported in experimental models and in patients with CI-AKI [35]. The biomarker most investigated in the setting of CI-AKI is* NGAL*. NGAL, a ubiquitous 25 KDa protein, covalently bound to gelatinase from human neutrophils, is a marker of tubular injury [36–38]. Serum NGAL (sNGAL) and/or urine NGAL (uNGAL) levels have been shown to predict AKI in different clinical settings [39–42], including CI-AKI [43–45]. Overall, NGAL was found to be a potentially useful tool for both the early (within few hours) diagnosis and prognosis (prediction of renal replacement therapy initiation and in-hospital mortality) of AKI [46, 47]. A typical example of the kinetic of sNGAL (markers of kidney damage), sCr, and sCyC (markers of functional change) is represented in Figure 2. The performance of NGAL (as for other kidney damage biomarkers) might depend on the different clinical setting. At present, limited evidence exists on (a) the optimal dosing site (urine versus blood) and time, (b) the cutoff value (or threshold), and (c) the clinical and prognostic significance of this kidney injury biomarker in the setting of CI-AKI. Recent systematic review and meta-analysis support that the diagnostic accuracy of plasma/serum NGAL (sNGAL) is similar to that of urine NGAL (uNGAL) [46]. uNGAL originates predominantly from kidney epithelia [48]. Thus, uNGAL expression appears specifically in distal tubular segments of injured nephrons, and it is not expressed in nonischemic zone [38, 48]. sNGAL might predominantly arise from the injured thick ascending tubules and the collecting ducts via back-leak form injured renal tissue, again reflecting renal damage [47, 48]. Also sNGAL may reveal the effect of toxins that injure multiple organs, besides kidney [48]. The concentration of urinary biomarkers of AKI is influenced by variation in urinary concentration within and between individuals. Normalizing to urine creatinine is usually used to account for variations in water reabsorption [49]. However, we did not observe any significant advantages when analyzing normalized uNGAL instead of the absolute uNGAL. This result is in agreement with the recent observation that for all injury biomarkers, absolute concentration performed best in the diagnosis of AKI [50].

It has been reported that NGAL increase (as index of kidney damage) is an independent predictor of unfavorable outcome, irrespective of the presence or not of functional damage [7]. It has been suggested that the real gold standard for the AKI biomarkers is whether they can be used to define and risk-stratify AKI and related complications, facilitating early diagnosis and interventions to improve clinical outcomes [47, 51]. Several studies suggest an analogy between the troponin/creatine kinase and the NGAL/creatinine relationship with a novel, more sensitive biomarker identifying previously undetected organ injury. Acute tubular damage might occur without detectable loss of excretory function and might predict worse clinical outcome. Therefore, NGAL and sCr reflect distinct pathophysiological events. Haase et al. recently demonstrated that, without diagnostic increase in sCr, NGAL positive patients might have likely subclinical AKI and carry a worse prognosis than NGAL negative patients [47]. Of note, the subset of patients with positive NGAL (NGAL+) had a similar adverse outcome to that observed in patients with positive creatinine (sCr+). NGAL+/sCr negative condition identified approximately 40% more AKI cases than sCr+ alone [7]. uNGAL was highly predictive of clinical outcomes, including nephrology consultation, renal replacement therapy, and admission to the intensive care unit [52]. Detection of elevated sNGAL might enable more rapid conventional interventions or introduction of novel therapies to prevent or effectively treat such otherwise undetected AKI [53].

*Conclusions.* The new biomarkers of CI-AKI have an important role in the early diagnosis of contrast-induced kidney damage. In the setting, NGAL seems to be the ideal biomarker. At present, open issues are (1) the optimal dosing site (urine versus blood) and time, (2) the cutoff value (or threshold), and (3) the clinical and prognostic significance of this kidney injury biomarker in the setting of CI-AKI. Further studies are warranted for clarifying these issues and, therefore, justify the adoption of the new biomarker (NGAL) in the routine clinical practice.

## Figures and Tables

**Figure 1:**
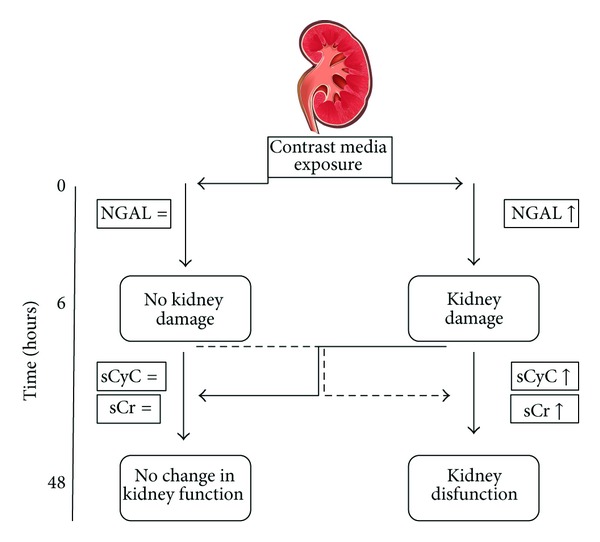
Phases of acute kidney injury. This figure illustrates progression from kidney damage (or injury) occurring after contrast media exposure to clinical changes in kidney function. The subclinical AKI occurs in few hours following contrast media exposure. This phase may be captured only by biomarkers of kidney damage (like neutrophil gelatinase-associated lipocalin (NGAL)) but not those of kidney function (like serum creatinine (sCr) or cystatin C (sCyC)). Kidney damage, in the majority of cases, remains subclinical (*subclinical AKI*). However, subclinical AKI may progress in the clinical phase, as defined by a deterioration of kidney function, detectable by the eventual (within 48 hours) increase in sCyC and/or sCr.

**Figure 2:**
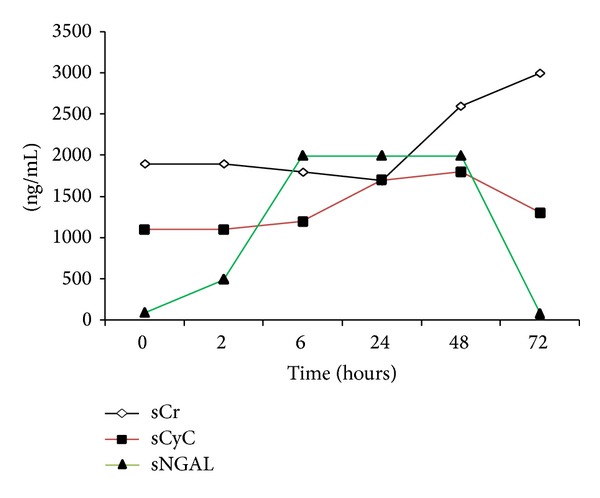
Kinetics of biomarkers during contrast-induced AKI. An example of CI-AKI is represented. Serum creatinine (sCr) (the “golden standard” of kidney function) typically raises at 48–72 hours after contrast media exposure. Serum cystatin C (sCyC) (a more sensitive marker of kidney function) raises within 24 hours after contrast media exposure. Serum neutrophil gelatinase-associated lipocalin (NGAL) (a marker of kidney damage) starts to raise at 6 hours after contrast media exposure.

**Table 1:** AKI biomarkers categories.

Inflammatory biomarkers:	
(i) neutrophil gelatinase-associated lipocalin (NGAL)	
(ii) interleukin-18 (IL-18)	

Tubular proteins:	
(i) kidney injury molecule-1 (KIM-1)	
(ii) Na+/H+ exchanger isoform 3 (NHE3)	

Surrogate markers of tubular injury:	
(i) urinary low molecular weight proteins escaping reabsorption on tubular injury (cystatin C, *β*2 or *α*1 microglobulin, and retinol binding protein)	
(ii) urinary tubular enzymes released on tubular injury (NAG [N-acetyl-*β*-D-glucosaminidase], alkaline phosphatase [AP], *γ*GT [gamma-glutamyl-transferase], etc.)	

**Table 2:** Ideal marker of contrast-induced acute kidney injury.

Easy to measure	
Does not require administration of an exogenous substance	
Sensitive to change	
Rapid change following injury	
Preferably specific to contrast injury	
A valid surrogate for clinically important outcomes (e.g., likelihood of needing dialysis, death, etc.)	
